# Theoretical Insights into Different Complexation Modes of Dioxovanadium(V) Compounds with Pyridoxal Semicarbazone/Thiosemicarbazone/S-Methyl-iso-thiosemicarbazone Ligands

**DOI:** 10.3390/molecules29061213

**Published:** 2024-03-08

**Authors:** Odeh Abdullah Odeh Alshammari, Sawsan Maisara, Badriah Alshammari, Maha Raghyan Alshammari, Violeta Rakic, Jasmina Dimitrić Marković, Violeta Jevtovic, Dušan Dimić

**Affiliations:** 1 Department of Chemistry, College of Science, University of Ha’il, Ha’il 81451, Saudi Arabia; 2 Department of Agriculture and Food Technology Prokuplje, Academy of Vocational Studies of South Serbia, 18400 Prokuplje, Serbia; 3 Faculty of Physical Chemistry, University of Belgrade, 11000 Belgrade, Serbia

**Keywords:** dioxovanadium(V), pyridoxal semicarbazone, pyridoxal isothiosemicarbazone, DFT, QTAIM, molecular docking, NBO

## Abstract

Vanadium complexes have gained considerable attention as biologically active compounds. In this contribution, three previously reported dioxovanadium(V) complexes with pyridoxal semicarbazone, thiosemicarbazone, and S-methyl-iso-thiosemicarbazone ligands are theoretically examined. The intermolecular stabilization interactions within crystallographic structures were investigated by Hirshfeld surface analysis. These experimental structures were optimized at the B3LYP-D3BJ/6-311++G(d,p)(H,C,N,O,S)/def2-TZVP(V) level of theory, and crystallographic and optimized bond lengths and angles were compared. High correlation coefficients and low mean absolute errors between these two data sets proved that the selected level of theory was appropriate for the description of the system. The changes in structures and stability were examined by adding explicit solvent molecules. The Quantum Theory of Atoms in Molecules (QTAIM) was employed to analyze the intramolecular interactions with special emphasis on the effect of substituents. A good correlation between electron density/Laplacian and interatomic distance was found. Through molecular docking simulations towards Bovine Serum Albumin (BSA), the binding affinity of complexes was further investigated. The spontaneity of binding in the active position of BSA was shown. Further experimental studies on this class of compounds are advised.

## 1. Introduction

Vanadium is a transition metal that is widespread in the soil. It is a trace element in many living beings, although there are examples of organisms in which vanadium is part of enzymes, such as vanadium nitrogenase and vanadium haloperoxidase. This metal has a broad range of oxidation states (from −3 to +5) that are easily interconverted [1]. The multiple oxidation states, good oxophillicity, and abundance make this metal and its compounds attractive in modern medicinal chemistry research [2]. The toxicity of vanadium depends on the form, oxidation state, route of intoxication, and dosage [3,4].

Vanadium compounds have been proposed for treating diabetes, cancer, and diseases caused by parasites [5,6,7]. Complexes containing vanadium belong to the non-platinum metal antitumor agents, offering an alternative to cancer chemotherapy [6]. Interest in these complexes has grown rapidly in the past 30 years, primarily because they have properties similar to phosphates [1,8]. It has been shown that these compounds interact with DNA and free radicals, inhibiting a cell cycle [9]. Other cancer pathways, such as MAPK/ERK and PI3K/AKT, can be targeted by vanadium-containing compounds [10]. In the review paper by Pessoa et al. [11], the possible application areas of vanadium in medicine are given. Antibacterial activity, cytotoxicity, hemolytic activity, and in vitro anticancer activity of synthesized vanadium complexes with 2-mercapto-5-methyl-benzimidazole as sulfur donor ligand are presented in reference [12]. In 2022, vanadium(IV) complexes were [13] synthesized from (*E*)-2-(((2-((2-hydroxyethyl)amino)quinolin-3-yl)methylene)amino)ethan-1-ol ligand (L) and vanadyl(IV) sulphate in methanol solutions. The complexes showed a considerable level of biological activity, while the quantum molecular descriptors from DFT calculations further supported the experimental results. In the paper by Maia and co-workers, the vanadium complexes containing thiosemicarbazone ligands showed higher activity towards *Mycobacterium tuberculosis* than free ligands [7].

**Figure 1:**
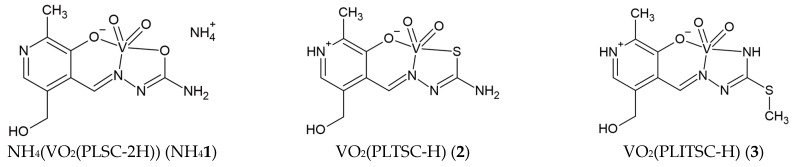
Molecular structure of different dioxovanadium(V) complexes included in the study.

The dioxovanadium(V) complexes with pyridoxal semicarbazone (PLSC) (CCDC code: HAHSIU), pyridoxal thiosemicarbazone (PLTSC) (CCDC code: OZITOI), and S-methyl-iso-thiosemicarbazone (PLITSC) (CCDC code: HAGWOD) were previously obtained and chemically characterized [14,15,16]. The structures of these complexes (NH_4_[VO_2_(PLSC–2H)], VO_2_(PLTSC–H)], [VO_2_(PLITSC–H)]) are presented in Figure 1. Complexes with similar ligands to those shown below have proven to be suitable catalysts for sulfoxidation [17,18] and selective oxidation of benzylic alcohols [19].

In presented structures, the central metal ion of vanadium is pentacoordinated, with two sites occupied by oxygen atoms, while the remaining three include donor atoms of the mentioned tridentate ligands (PLSC, PLTSC, and PLITSC, respectively). The first complex ion is anionic, while the other two are neutral. This is a consequence of the protonation/deprotonation of ligands, as presented in Figure 1. The synthesis of all three complexes was also very similar, with the starting salt being NH_4_VO_3_, which, in a water-methanol solution with the added ammonia, reacted with the ligand via reflux for a couple of hours. The color of the obtained complexes is orange, which is attributed to vanadium(V) ions. Only the vanadium complex NH_4_[VO_2_(PLSC–2H)] [4] was studied for biological activity, while for the others, only the synthesis and crystal structure were reported.

In this contribution, the stability and protein binding affinity of these complexes are examined by theoretical methods. The intermolecular interactions within the crystallographic structure were assessed by Hirshfeld surface analysis, and the change in percentages of specific contacts was explained by the present ending donor atoms. The crystallographic structures were optimized at the B3LYP-D3BJ/6-311++G(d,p)(H,C,N,O,S)/def2-TZVP(V) level of theory, and the changes in bond lengths and angles were discussed. The Quantum Theory of Atoms in Molecules (QTAIM) was used to analyze the interactions between various donor atoms and vanadium ions. The affinity of three complexes towards Bovine Serum Albumin, a model transport protein, was examined by the molecular docking simulations, and the effects of ligand structure were outlined.

## 2. Results and Discussion

### 2.1. Hirshfeld Surface Analysis of Crystallographic Data

**Figure 2:**
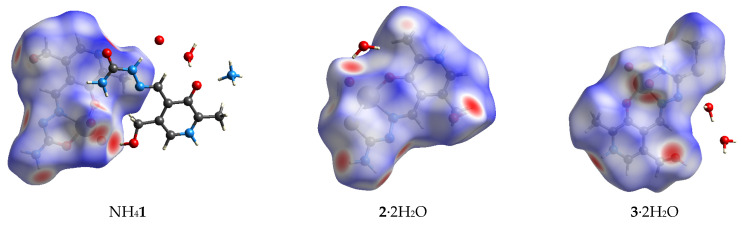
Hirshfeld surfaces of different dioxovanadium(V) complexes included in the study.

Hirsfeld surface analysis is important for examining the intermolecular interactions within crystallographic structures. The structures of three examined dioxovanadium(V) compounds possess a certain degree of similarity where structural features are concerned. The complex anion **1** is negatively charged due to the deprotonation of two oxygen atoms. In this compound, dioxovanadium(V) is coordinated to an aromatic oxygen atom, hydrazine nitrogen, and an oxygen atom attached to an aliphatic chain. Complexes **2** and **3** are neutral, with ligands being singly deprotonated. In compound **2,** the coordination sphere has oxygen atoms exchanged with sulfur. The aliphatic chain differs for the last compound as the ending group is rotated compared to **2,** and a methyl group is added to a sulfur atom. Therefore, these changes can influence the type and percentages of specific contacts within a crystallographic structure. Figure 2 shows the Hirshfeld surfaces of investigated compounds, and the fingerprint plots for the most numerous contacts are presented in Figures S1–S3. Table 1 represents the percentages of the most numerous contacts. It is crucial to outline that only complex ions or neutral compounds containing dioxovanadium(V) are included in examining the formed interactions to determine structural effects.

The central metal ion of vanadium is included in a limited number of interactions with neighboring atoms (V∙∙∙C (**1** and **3**, 0.4%) and V∙∙∙H (**2**, 0.5%)) (Table 1). The most numerous interactions include only hydrogen atoms (H∙∙∙H). Their percentages are very similar among the three investigated compounds (30.6 (**1**), 25.6 (**2**), and 33.9% (**3**)). The highest percentage of interactions for compound **3** can be expected due to the relative flexibility of the methyl group attached to a sulfur atom. The hydrogen bonds are the most important interactions for the stabilization of structure. The O∙∙∙H contacts have the highest percentage of appearance in the case of **1** (40%), followed by **2** (33.8%) and **3** (30.2%). Oxygen as a donor atom in compound **1** increases the percentage of these contacts. It should be kept in mind that other compounds within the crystal package influence these values, such as water molecules and neutral ligands. The same trend can be observed when the nitrogen atom is concerned. When nitrogen atoms of the ending amino group are not included in interactions with vanadium, the contact denoted as N∙∙∙H has percentages of 11.3 and 11.5% for **1** and **2**. This amount is lowered to 10.1% when a donation of a lone pair to vanadium occurs in **3**. The interactions between carbon and hydrogen atoms are between 9.7 (**2**) and 5.7% (**3**). These interactions also include contacts between positively charged hydrogen atoms and the π-electron cloud of the aromatic ring.

The interactions between electronegative atoms are strongly influenced by the present substituents and binding mode to dioxovanadium(V) moiety. For example, O∙∙∙O interactions were found only in **2** (1.1%), while O∙∙∙N contacts were present in **1** (0.8%) and **3** (0.6%), similar to other compounds [18]. The interactions between nitrogen and carbon atoms were present between 5.5 (**1**) and 2.2% (**2**). The O∙∙∙C contacts have almost equal percentages in all compounds (~3.2%). The introduction of sulfur atoms in ligands PLTSC and PLITSC led to novel interactions with oxygen (1.9 (**2**) and 1.5% (**3**)), hydrogen (9.7% (**2**)), and carbon (1.1% (**3**)) atoms (Table 1). Once the sulfur atoms are included in binding mode with the vanadium central ion, the interactions between this electronegative element and the hydrogen atom of surrounding solvent molecules can be observed. When a sulfur atom is part of a routable chain ending, interactions with carbon atoms of neighboring ligands are present. The effect of geometry and stabilization interactions depending on the position of substituents are examined in more detail in the following section.

### 2.2. Optimization of Structures and Comparison with Crystalographic Data

**Figure 3:**
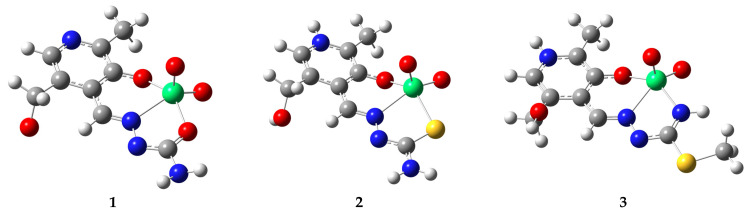
Optimized structures of different dioxovanadium(V) complexes included in the study (at B3LYP-D3BJ/6-311++G(d,p)(H,C,N,O,S)/def2-TZVP(V) level of theory) (Hydrogen—white, carbon—grey, nitrogen—blue, oxygen—red, vanadium—green).

The optimization of the crystallographic structures was performed without any geometrical constraints at the B3LYP-D3BJ/6-311++G(d,p)(H,C,N,O,S)/def2-TZVP(V) level of theory. The experimental and theoretical bond lengths and angles were compared by calculating the correlation coefficient (R) and the mean absolute error (MAE). The second parameter calculates the average value of the absolute difference between experimental and theoretical bond lengths and angles. These two data sets are presented in Tables S1–S6, while the optimized structures are shown in Figure 3. The optimized structures are needed for further analysis of the stabilization interactions and possible affinity towards transport proteins. Only non-hydrogen atoms are included in this analysis.

When experimental and optimized bond lengths were compared, the correlation coefficients between 0.994 (**1** and **2**) and 0.996 (**3**) were obtained (Tables S1, S3 and S5). The average differences between these two data sets were of the experimental error, around 0.02 Å. The distances between vanadium ion and oxygen atoms directly attached in dioxovanadium(V) moiety are between 1.62 and 1.65 Å in experimental and 1.60 and 1.61 Å in theoretical structures. Upon optimization, these bond lengths equilibrated. It is important to outline that these bond lengths were not changed depending on the other donor atoms. The bond length between the aromatic oxygen atom and vanadium was 1.90 and 1.92 Å in complexes **2** and **3,** and 1.86 Å in complex **1**. This change in bond length was probably a consequence of the other interactions with surrounding atoms [19]. The bond distance between hydrazine nitrogen and vanadium is also in a narrow range between 2.15 and 2.20 Å in experimental and between 2.26 and 2.35 Å in theoretical structures. Depending on the present substituent on the aliphatic chain, specific changes in electron delocalization are possible, influencing the donation of electrons to vanadium. The most significant differences in bond lengths were observed for the changing donor atom. In experimental/optimized structures, the following bond lengths were found: 1.95/2.03 for O−V (**1**), 2.35/2.41 for S−V (**2**), and 2.00/2.04 Å for N−V (**3**), which are consistent with previously reported data [16]. These values nicely follow the electronegativity change of the donor atoms; further insight into these interactions is presented in the following section. The other bond lengths are within the expected range and do not change significantly upon the change in substituent, as examined previously within our research group [20,21,22,23].

The changes in bond angles are more pronounced than bond lengths due to the overall relaxation of the system during optimization. These calculations were performed for an isolated molecule in a vacuum, while other compounds can be present in a crystallographic structure, as shown in Figure 2. The correlation coefficients are between 0.977 (2) and 0.993 (1), while MAE values are between 1.3 and 2.5°. The most notable differences between the two data sets were observed for the groups surrounding the central metal ion. In each of the three complexes, the geometry around the central metal ion is distorted square pyramidal, a common geometry for vanadium(V) complexes [16]. One of the oxygen atoms in all complexes is at the top of the pyramid, while the other four form a foundation. For complex 1, the differences between experimental and theoretical bond angles around the central metal ion are between 0.7 and 4.6°. This most significant difference covers the angle between two oxygen atoms of the PLSC ligand and the central metal ion as part of the overall relaxation of structure and adjustment of bonds when no other compounds are present to stabilize structure through specific interactions. A difference of 8° was found for the angle formed between the aromatic oxygen atom, vanadium, and sulfur within structure **2,** which could be explained by the relaxation of the ligand structure. An additional reason is that water molecules from the crystallographic structure (Figure 2) were omitted from optimization, which could be necessary for the system’s overall stability [16]. Therefore, it is recommended to occasionally include co-crystalized solvent molecules as they affect the system, primarily when spectral prediction is performed. The differences in bond angles of the atoms surrounding the central metal ion of complex **3** are between 0.8 and 4.5°. Based on the structural comparison, it can be concluded that the optimized structures represent the experimental ones well and that the selected level of theory is appropriate for further investigation of the stabilization of intramolecular interactions.

The NBO analysis is useful for investigating the strength of bonding in metal complexes. This method of calculating the atomic charges is less sensitive to the basis set variation when compared to Mulliken charge populations. The net atomic charges of vanadium ions are 0.724 (**1**), 0.500 (**2**), and 0.656 *e* (**3**). These charges are lower than formal charges +5, proving that significant electron density transfer from ligand to metal ions occurred [24]. The net charges of ligands PLSC, PLTSC, and PLITSC are −724, −0.500, and −0.656 *e*, respectively. It should be kept in mind that the total charge of **1** is −1. This leads to the conclusion that 0.276, 0.500, and 0.344 *e* of electron density are transferred to metal ions from ligands. When structural parameters are considered, it is clear that the presence of electronegative substituents and donor atoms greatly influences the electron density donation. This amount of electron density follows the decrease in electronegativity of oxygen, nitrogen, and sulfur atoms.

### 2.3. Explicit Solvent Effect Investigation

**Figure 4:**
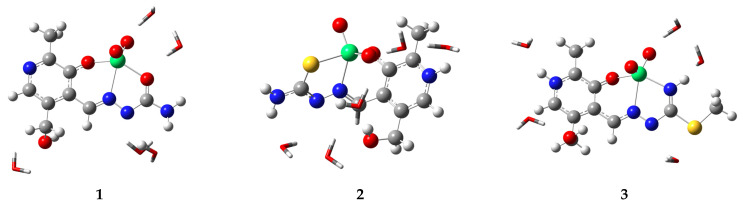
Optimized structure of **2** with five molecules of water (using semi-empirical PM6 method) (Hydrogen—white, carbon—grey, nitrogen—blue, oxygen—red, vanadium—green, water molecules represented by tube model).

The structures of complexes were reoptimized with five molecules of water to encounter the possible solvent effect on the structure and stability using the PM6 method. Optimized structures with solvent molecules are depicted in Figure 4. Due to several polar groups, the solvent molecules form specific solvent–solute interactions. As presented, hydrogen bonds were formed between protonated pyridine nitrogen, protonated amino nitrogen, hydroxymethyl oxygen, and oxygen atoms from the dioxovanadium(V) moiety. Additional interactions between two water molecules can also be found. A certain distortion in the complex geometry was shown due to these interactions.

Upon optimization, the distances between oxygen and vanadium atoms in the dioxovanadium(V) moiety are in a broader range (1.57–1.64 Å) as several hydrogen bonds are formed between these oxygen atoms and surrounding water molecules. The bond lengths between the aromatic oxygen atom and vanadium are 1.91 (1), 2.05 (2), and 1.97 Å (3, which on average differs for 0.05 Å as part of the relaxation of structure. No significant differences for the bonds between hydrazine nitrogen atoms and vanadium ions were found. The bond lengths for V–X are 2.09 (V–O), 2.37 (V–S), and 2.13 Å (V–N), which is comparable to the previous findings. It is important to outline that the structure of complex **2** is distorted the most when bond angles are concerned due to the weakest interaction between vanadium and sulfur atoms, as shown previously. In the optimized structures with five molecules of water, additional separation of charges occurred. The changes in vanadium ions are 0.831 (**1**), 0.551 (**2**), and 0.770 *e* (**3**). These values are still lower than +5, which is the formal charge on this ion, probably because of the electron density transfer from ligands and water molecules. The charge on vanadium ions still follows the electronegativity trend of oxygen, sulfur, and nitrogen atoms. The change in charge of the central metal ion is more pronounced in the case of oxygen and nitrogen donor atoms. Therefore, it can be concluded that including explicit solvent molecules profoundly affects the stability of structures, intramolecular interactions, and spectra of investigated compounds.

### 2.4. QTAIM Analysis of Structures

The QTAIM analysis of the intramolecular interactions is used to quantify these interactions. Table 2 shows the electron density and Laplacian for the Bond Critical Point between the donor atoms and vanadium ions. The parameters for V=O bonds are given separately for the oxygen atom in a plane with other donor atoms (plane) and at the top of the structure (top).

The QTAIM parameters are correlated to the bond distance for the donor–acceptor interactions within the structures in Table 2. Based on these values, several distinct regions can be outlined. The strongest interactions are formed between oxygen and vanadium atoms within dioxovanadium(V) moiety, with the values of electron density and Laplacian on average being 0.269 and 0.926 a.u. These values prove the previous assumption that the present substituent does not influence these bonds, also reflected in the bond length of 1.61 Å. The second group of interactions includes those with aromatic oxygen atoms, and oxygen and nitrogen atoms of ending groups on an aliphatic chain in complexes **1** and **3**. The bonds have lengths between 1.93 and 2.04 Å. Again, the QTAIM parameters of the interaction between aromatic oxygen and vanadium are not dependent on the other substituents. The oxygen and nitrogen atoms in the position of substituents have almost the same values of electron density (0.080/0.090 a.u.) and Laplacian (0.380/0.299 a.u.), which proves that these atoms similarly interact with the central metal ion. The longest distances were observed for the interactions, including hydrazine nitrogen and sulfur atoms. The bond lengths between hydrazine nitrogen and vanadium ions are between 2.26 and 2.35 Å and depend on the system’s interaction strength with other donor atoms (O,S,N). The distance between V and S is 2.41 Å, with the lowest values of the QTAIM parameters. It has been discussed previously that the elongation of the V–N_hydrazine_ bond (2.29 Å) is due to the trans effect of the basal oxo O ligand, which is stronger than the trans effect of aromatic oxygen exerted on the V–N_amine_ bond (2.04 Å) [16]. These results have shown that QTAIM parameters can be used to predict the stabilization effects of different donor atoms in similar ligand systems.

The QTAIM parameters can be used to discuss the ionic/covalent nature of interactions. In this contribution, this examination is limited to the V–X (X=O, S, N) bonds as they are different between these complexes. The classification of bonds was proposed by Bianchi and coworkers [25] based on the ratio between absolute values of potential energy density (*V_BCP_*) and kinetic energy density (*G_BCP_*). If the ratio *V_BCP_/G_BCP_* is higher than 2, the bond can be classified as covalent, while a bond characterized by values between 1 and 2 can be considered ionic with a small amount of covalency and dative or coordinate bonds. The ratios between these parameters are 1.037 (V–O), 1.340 (V–S), and 1.187 (V–N). All of the investigated bonds have a partial ionic and covalent character. As shown above, the degree of covalent character decreases, which is well reflected in the bond length and electronegativity in present atoms. With the increase in electronegativity of donor atoms, the V–X bonds are shorter with higher ionic character.

### 2.5. Molecular Docking Study of Interactions with BSA

The molecular docking simulations were performed towards BSA as a main transport protein model used to investigate compounds’ distribution potential. The binding was localized around the active pocket, including TRP213 amino acid, commonly accessed by spectrofluorometric titration [26,27,28]. The most stable conformations of investigated compounds are presented in Figure 4, while the contributions to the binding energies are listed in Table S7.

**Figure 5:**
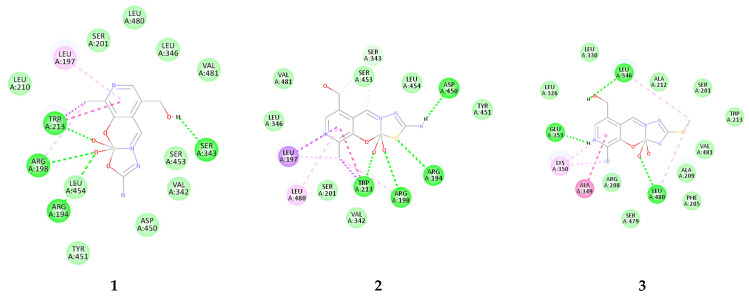
The most stable configurations of investigated complexes in the active pocket of BSA. (van der Waals/carbon–hydrogen bonds—light green, conventional hydrogen bonds—dark green, π-σ/stacking—dark pink, alkyl/π-alkyl—light pink).

The results from Table S7 prove that all three compounds bind to the active pocket of BSA spontaneously, with the binding energy of −15.8, −19.3, and −16.7 kJ mol^−1^ for complexes **1**, **2**, and **3**, respectively. These values are comparable to the experimentally obtained change in Gibbs free energy of binding between BSA and vanadium(IV) complexes of salicylaldehyde-based furoic acid hydrazones [29]. The main contribution to these energies comes from the weak interactions, including van der Waals and hydrogen bonds. The electrostatic energy is the highest for **3** (−4.1 kJ mol^−1^), while it is the lowest for **1** (−2.1 kJ mol^−1^). The torsional energies are the same for all three compounds, showing that rotation is not dependent on the present substituents. A closer look into the binding positions can be found in Figure 5. All compounds are bound in the vicinity of TRP213, which is consistent with experimental studies from reference [30]. Complex **1** forms four conventional hydrogen bonds through electronegative groups. The OH group interacts with SER343 (1.81 Å), while oxygen atoms from the dioxo-moiety form hydrogen bonds with TRP213 (1.67 Å), ARG198 (3.30 Å), and ARG194 (2.46 Å). Other amino acids are included in weak interactions, as shown in Figure 4. In the case of **2**, the ending amino group forms a conventional hydrogen bond with ASP450 (1.92 Å), sulfur with ARG194 (2.73 Å), while two oxygen atoms interact with TRP213 (1.64 Å) and ARG198 (2.59 Å). There is an additional π-alkyl interaction with LEU194. The most stable conformation of **3** is bound in slightly different surroundings. The OH group forms a hydrogen bond with LEU346 (2.24 Å), protonated nitrogen with GLU353 (1.71 Å), and oxygen with LEU480 (2.35 Å). This complex also interacts with TRP213 through weak van der Waals interactions. When all of these structures are compared, it is important to conclude that the abundance of electronegative groups is essential for the spontaneity of binding. Therefore, future experimental studies on the interactions between transport proteins and dioxovanadium(V) compounds with PLSC, PLTSC, and PLITSC are advised, as these results might shed light on the distribution of these compounds in the human body. A careful combination of the substituents allows fine-tuning binding affinities towards BSA.

## 3. Materials and Methods

### 3.1. Hirshfeld Surface Analysis

The stabilization interactions within the crystal structure are crucial for its stability. These interactions can be quantitatively measured through Hirshfeld surface analysis within the CrystalExplorer program [31] package based on the crystallographic structure. This analysis is represented by a graph connecting two distances: one that represents the distance between the two nearest nuclei (de) and the second between nuclei and the external surface (di) [32,33,34]. These values are normalized and colored depending on the corresponding van der Waals radii separation. Within this contribution, red, white, and blue colors represent shorter, equal, and longer separations than the respective van der Waals radii. Fingerprint plots are prepared for each type of contact and allow for determining the percentages of specific interactions. The crystallographic structures were taken from the Cambridge Crystallographic Data Centre, as found in the cited references.

### 3.2. Theoretical Analysis

The crystallographic structures of three dioxovanadium(V) complexes were taken from previous contributions of our research group. The optimizations were performed in the Gaussian 09 Program Package [35] starting from these experimental structures. The Global Hybrid Generalized Gradient Approximation (GGA) functional B3LYP [36] in conjunction with 6-311++G(d,p) basis set [37] for H, C, N, S, and O atoms and def2-TZVP basis set [38,39] for Va were applied for the optimization. The dispersion corrections were included through the D3BJ [40]. Previously, the same level of theory was used for other oxovanadium compounds [18,41,42]. The vibrational spectra were calculated alongside optimization, and the absence of imaginary frequencies was taken as proof that minima on the potential energy surface were found. The optimizations were performed without any geometrical constraints on complex ions, without the presence of counter ions and solvent molecules. The structures of complexes with five molecules of water were optimized using a semi-empirical PM6 model [43]. The Natural Bond Orbital charges were calculated within the NBO analysis [44] approach, as implemented in the Gaussian 09. The Quantum Theory of Atoms in Molecules (QTAIM) [45,46] was used to examine these interactions within the AIMAll package [47]. Within this analysis, there are two types of interactions depending on the values of electron density and Laplacian in the Bond Critical Points (BCP). Closed-shell interactions that include covalent bonds are characterized by the electron density of 0.1 a.u. and large negative Laplacian. Open-shell interactions, such as ionic bonds, hydrogen bonds, and van der Waals interactions, have an electron density between 0.001 and 0.04 a.u. and a small but positive Laplacian [48].

### 3.3. Molecular Docking Analysis

The effect of the geometry of the complex on its interactions with biomolecules was predicted by the molecular docking analysis towards BSA as an example of the transport protein commonly used in experiments. The AutoDock 4.2 software [49] calculates the binding affinity. The target crystal structure was obtained from the RCSB Protein Data Bank in PDB format (PDB: 4OR0) [50]. Before docking, the protein was prepared by removing co-crystalized ligands, water molecules, and cofactors. The AutoDockTools graphical user interface [51] was employed to calculate the Kollman partial charges and add polar hydrogen atoms. The Lamarckian Genetic Algorithm (LGA) [52] was used to perform protein–ligand flexible docking. The search space for the most stable conformation of compounds was performed in a grid box with dimensions 60 × 60 × 60 Å and a grid spacing of 0.375 Å. The active position was localized around TRP213 (IIA, −1.0, 21, and 119 Å). From these calculations, different contributions to the binding energy can be determined through the following equation:

Δ**G**_bind_
*=* Δ**G**_vdw+hbond+desolv_ *+* Δ**G**_elec_
*+* Δ**G**_total_
*+* Δ**G**_tor_ − Δ**G**_unb_ (1)

where Δ**G**_bind_ is the estimated free energy of binding, and Δ**G**_vdw+hbond+desolv_ denotes the sum of the energies of the dispersion and repulsion (Δ**G**_vdw_), hydrogen bond (Δ**G**_hbond_), and desolvation (**ΔG**_desolv_). Δ**G**_total_ represents the final total internal energy, and Δ**G**_tor_ is torsional free energy, Δ**G**_unb_ is the unbound system’s energy, and Δ**G**_elec_ is electrostatic energy. The BIOVIA Discovery studio [53] was used to evaluate the results and visual representation of interactions within the active pocket.

## 4. Conclusions

The dominant contacts within crystallographic structures of the investigated compounds were H∙∙∙H (between 25.6 (**2**) and 33.9% (**3**)), followed by O∙∙∙H contacts. The present substituents had a pronounced effect on these percentages, especially where contacts that include nitrogen and sulfur atoms are concerned. The structures optimized at the B3LYP-D3BJ/6-311++G(d,p)(H,C,N,O,S)/def2-TZVP(V) level of theory showed a high resemblance with the experimental ones. The correlation coefficients were between 0.994 and 0.996 for bond lengths and between 0.977 and 0.993 for bond angles. The MAE values for bond lengths were of the order of experimental error, while for bond angles these values were higher due to the system’s relaxation. The importance of co-crystalized solvent molecules and counter-ions was discussed. Differences in bond lengths and charge separation were found upon the addition of explicit solvent molecules. When QTAIM parameters were concerned, there were three distinct regions depending on the strength of interactions and interatomic distance. All three complexes spontaneously bound in the active pocket of BSA near TRP213. The main contributions to these interactions are conventional hydrogen bonds with surrounding amino acids. The oxygen atoms of dioxo-moiety are critical structural parameters for protein binding. Further experimental studies on interactions with the transport protein are advised to elucidate additional critical structural features for this process.

## Figures and Tables

**Table 1:** The percentages of the most important contacts in the crystallographic structures determined by Hirshfeld surface analysis.

Contact	1	2	3
V∙∙∙C	0.4	/	0.4
V∙∙∙H	/	0.5	/
H∙∙∙H	30.6	25.6	33.9
H∙∙∙O	40.0	33.8	30.2
H∙∙∙N	11.3	11.5	10.1
H∙∙∙C	5.8	9.7	5.7
H∙∙∙S	/	9.7	/
O∙∙∙N	0.8	/	0.6
O∙∙∙C	3.2	/	3.4
O∙∙∙O	/	1.1	/
O∙∙∙S	/	1.9	1.5
N∙∙∙N	0.9	1.0	/
N∙∙∙C	5.5	2.2	4.0
C∙∙∙C	/	1.9	/
C∙∙∙S	/	/	1.1

**Table 2:** Electron density, Laplacian, and distance of the interactions between donor atoms and vanadium in investigated compounds.

Complex	Title 2	V=O (Plane/Top)	V–O_aromatic_	V–N_hydrazine_	V–X (O, S, N for 1, 2, and 3)
**1**	Electron Density [a.u.]	0.265/0.267	0.100	0.054	0.080
	Laplacian [a.u.]	0.925/0.912	0.482	0.196	0.380
	Distance [Å]	1.61/1.61	1.93	2.26	2.03
**2**	Electron Density [a.u.]	0.269/0.272	0.095	0.043	0.066
	Laplacian [a.u.]	0.942/0.921	0.474	0.162	0.118
	Distance [Å]	1.60/1.60	1.94	2.35	2.41
**3**	Electron Density [a.u.]	0.267/0.271	0.094	0.049	0.090
	Laplacian [a.u.]	0.935/0.922	0.470	0.183	0.299
	Distance [Å]	1.61/1.60	1.95	2.29	2.04

## Data Availability

Data are contained within the article and Supplementary Materials.

## Supplementary Materials

The following supporting information can be downloaded at: https://www.mdpi.com/article/10.3390/molecules29061213/s1, Figure S1: The fingerprint plots of the most important contacts in structure of **1**; Figure S2: The fingerprint plots of the most important contacts in structure of **2**; Figure S3: The fingerprint plots of the most important contacts in structure of **3**; Table S1. Experimental and theoretical (at B3LYP-D3BJ/6-311++G(d,p)(H,C,N,O)/def2-TZVP(V) level of theory) bond lengths of **1** (the notation follows the figure below); Table S2. Experimental and theoretical (at B3LYP-D3BJ/6-311++G(d,p)(H,C,N,O)/def2-TZVP(V) level of theory) bond angles of **1** (the notation follows the figure above); Table S3. Experimental and theoretical (at B3LYP-D3BJ/6-311++G(d,p)(H,C,N,O,S)/def2-TZVP(V) level of theory) bond lengths of **2** (the notation follows the figure below); Table S4. Experimental and theoretical (at B3LYP-D3BJ/6-311++G(d,p)(H,C,N,O,S)/def2-TZVP(V) level of theory) bond angles of **2** (the notation follows the figure above); Table S5. Experimental and theoretical (at B3LYP-D3BJ/6-311++G(d,p)(H,C,N,O,S)/def2-TZVP(V) level of theory) bond lengths of **3** (the notation follows the figure below); Table S6. Experimental and theoretical (at B3LYP-D3BJ/6-311++G(d,p)(H,C,N,O,S)/def2-TZVP(V) level of theory) bond angles of **3** (the notation follows the figure above); Table S7. The important thermodynamic parameters (in kJ mol^–1^) for the best docking conformation of the investigated complexes with BSA.
