# Supplementary material for: Theoretical Insights into Different Complexation Modes of Dioxovanadium(V) Compounds with Pyridoxal Semicarbazone/Thiosemicarbazone/S-Methyl-iso-thiosemicarbazone Ligands
Source: Molecules. 2024 Mar 8;29(6):1213. doi: 10.3390/molecules29061213 (PMC10976139; doi:10.3390/molecules29061213)
Supplement: Supplementary file 1 [file molecules-29-01213-s001.zip › molecules-2906786-supplementary.pdf]

Supplementary Information for:

Theoretical insights into different complexation modes of dioxovanadium(V) compounds with pyridoxal semicarbazone/thiosemicarbazone/S-methyl-iso-thiosemicarbazone ligands

Odeh Abdullah Odeh Alshammari [1], Sawsan Maisara [1], Violeta Rakic [2], Jasmina Dimitrić Marković [3], Violeta Jevtovic [1] and Dušan Dimić [3,*]

[1] Department of Chemistry, College of Science, University of Ha'il, Ha'il 81451, Saudi Arabia
[2] Department of Agriculture and Food Technology Prokuplje, Academy of Vocational Studies of South Serbia, 18400 Prokuplje, Serbia;
[3] Faculty of Physical Chemistry, University of Belgrade, 11000 Belgrade, Serbia
* Correspondence: v.jevtovic@uoh.edu.sa (V.J.); ddimic@ffh.bg.ac.rs (D.D.)

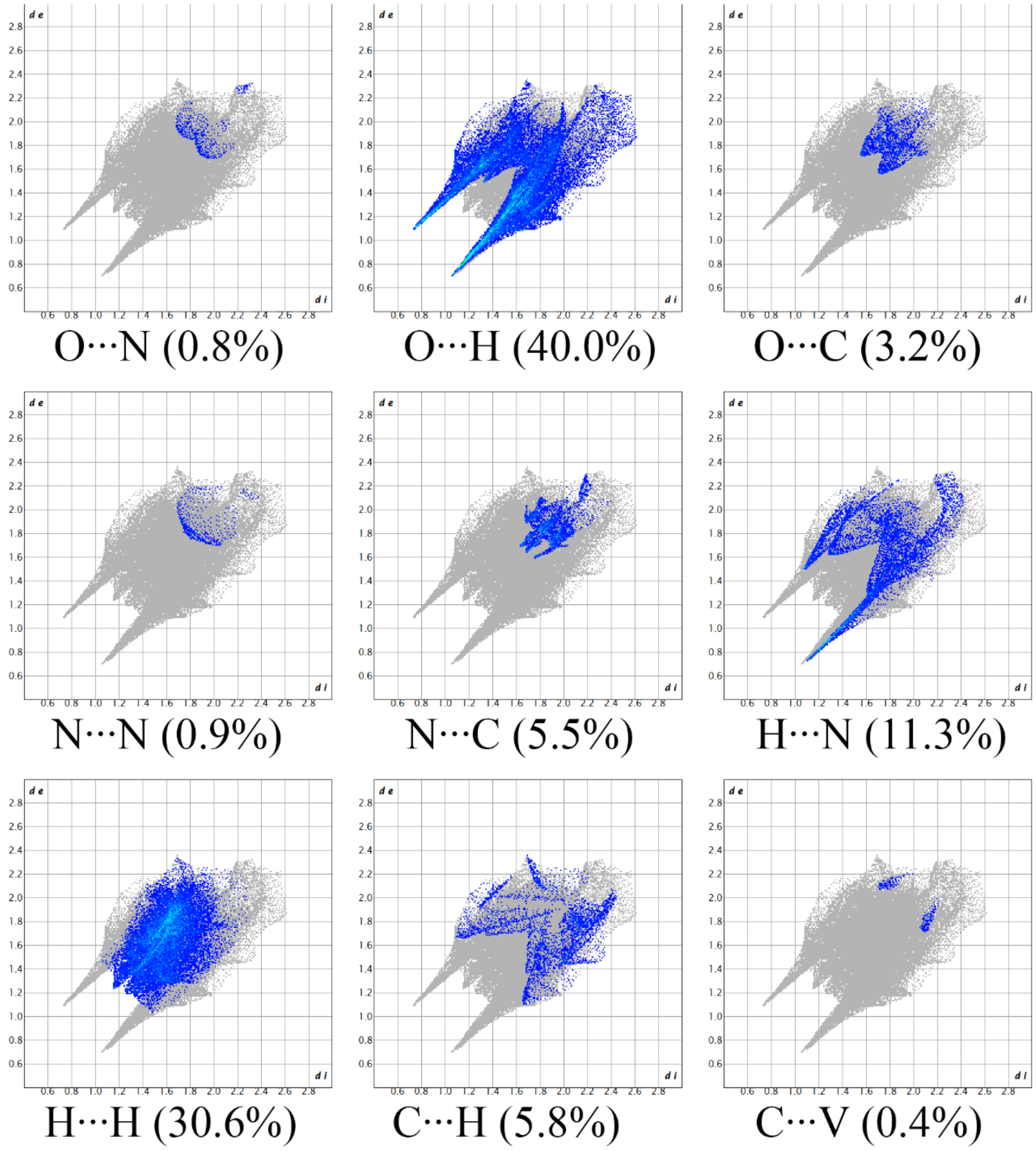

Figure S1. The fingerprint plots of the most important contacts in structure of **1**.

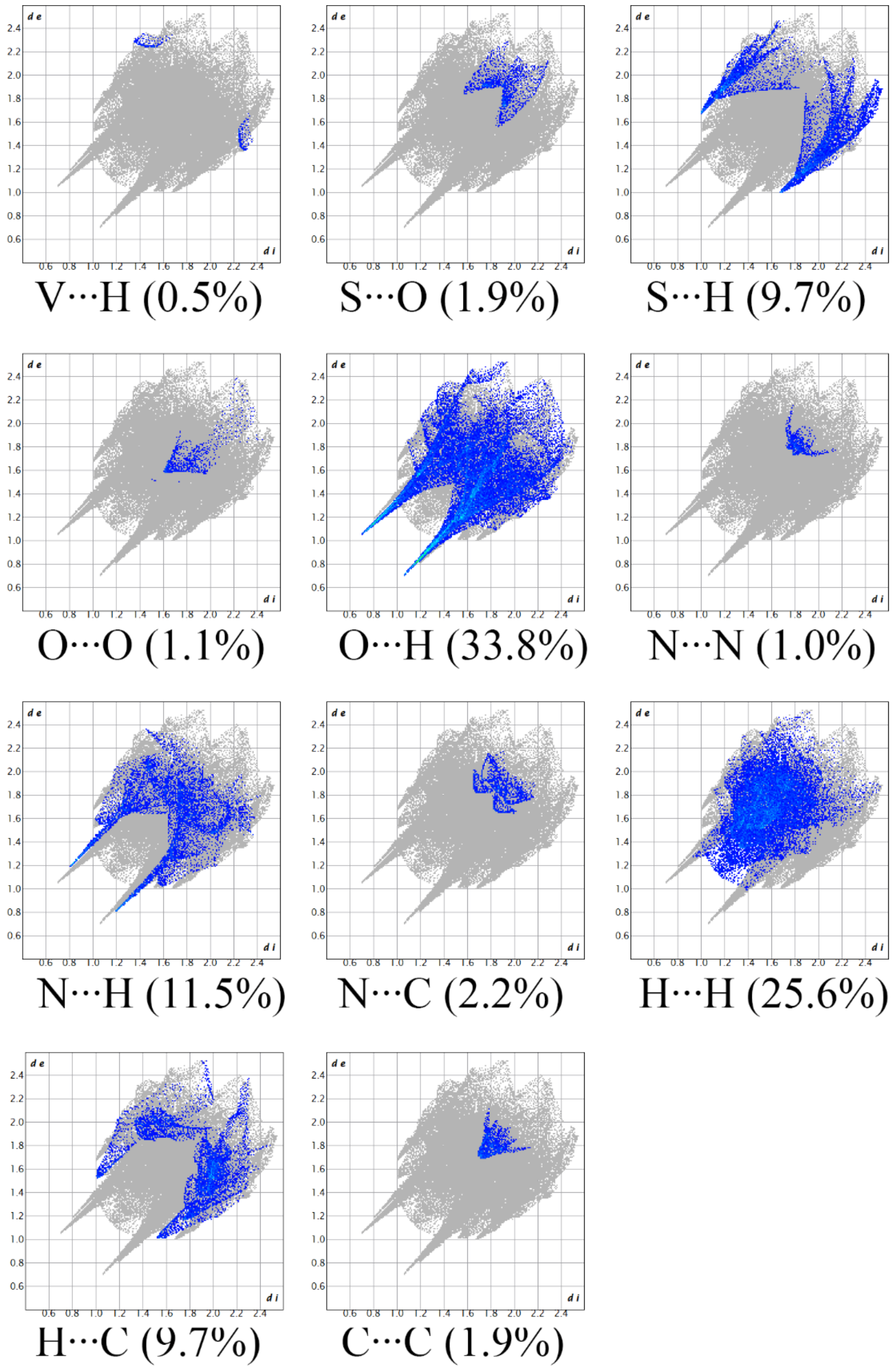

Figure S2. Fingerprint plots of the most important contacts in structure of **3**.

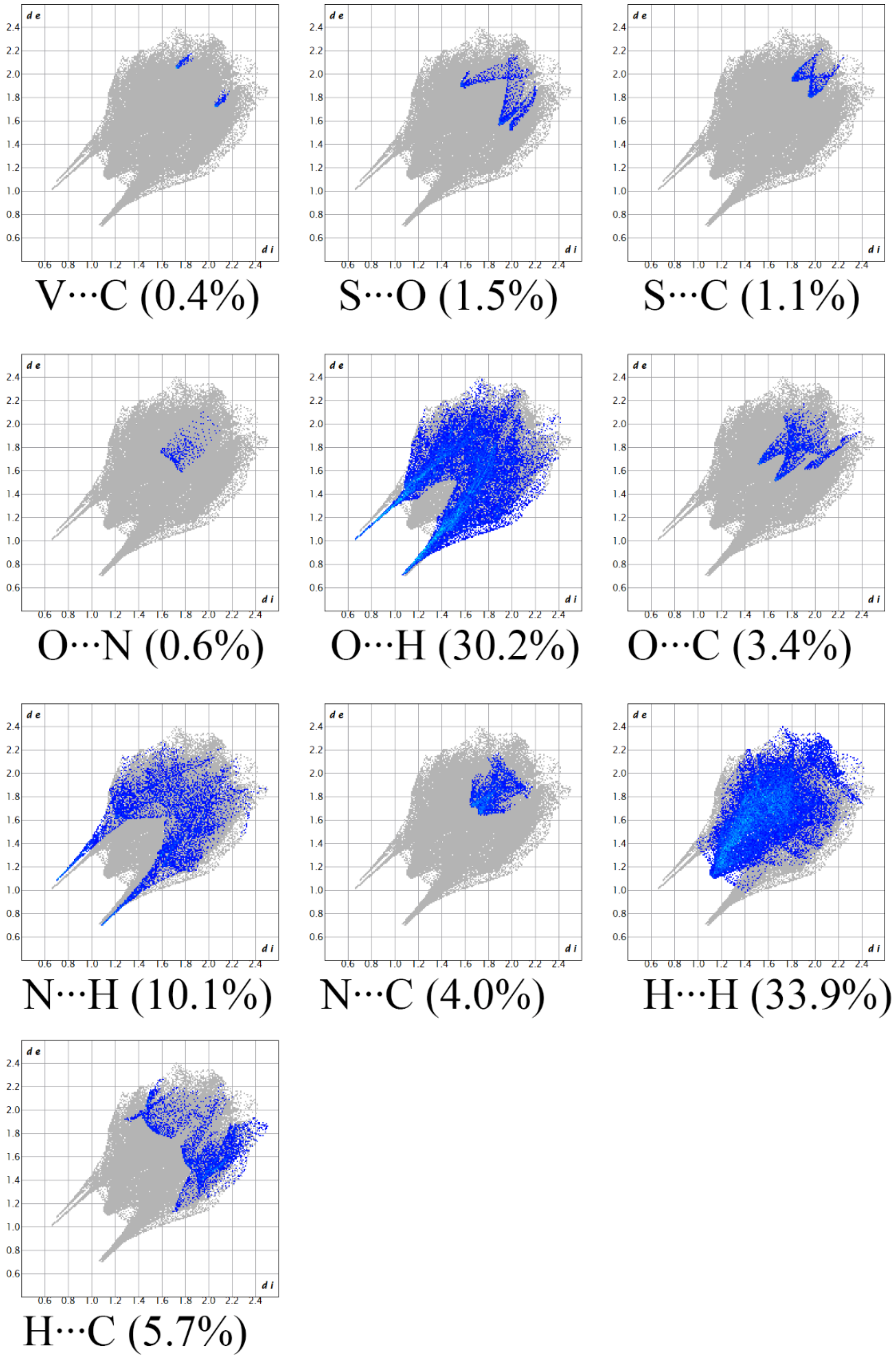

Figure S3. Fingerprint plots of the most important contacts in structure of **2**.

Table S1. Experimental and theoretical (at B3LYP-D3BJ/6-311++G(d,p)(H,C,N,O)/def2-TZVP(V) level of theory) bond lengths of **1** (the notation follows the figure below)

Bond	Experimental	Theoretical
V1-O2	1.65	1.61
V1-O3	1.86	1.93
V1-O4	1.95	2.03
V1-O5	1.62	1.61
V1-N11	2.15	2.26
O4-C23	1.31	1.28
C23-N8	1.32	1.38
C23-N13	1.32	1.32
N13-N11	1.38	1.37
N11-C15	1.29	1.29
C15-C14	1.44	1.44
C14-C17	1.39	1.42
C17-O3	1.32	1.31
C17-C22	1.41	1.43
C22-C18	1.49	1.50
N12-C22	1.33	1.33
N12-C25	1.35	1.34
C25-C24	1.37	1.39
C24-C27	1.50	1.50
C27-O6	1.43	1.44
C24-C14	1.41	1.42
R		0.994
MAE [Å]		0.02

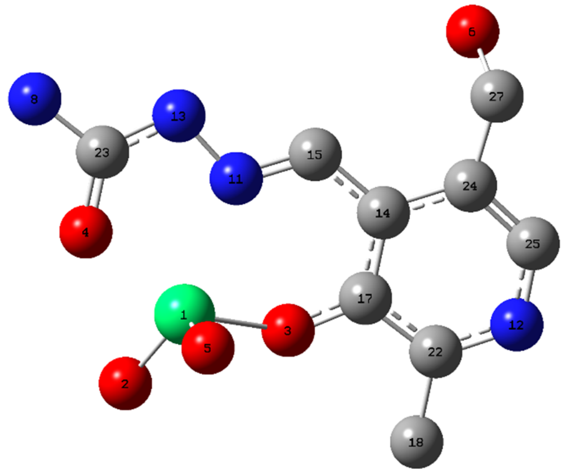

Table S2. Experimental and theoretical (at B3LYP-D3BJ/6-311++G(d,p)(H,C,N,O)/def2-TZVP(V) level of theory) bond angles of **1** (the notation follows the figure above)

Bond angle	Experimental	Theoretical
O2-V1-O3	96.6	97.6
O2-V1-O4	91.5	93.6
O2-Vi-O5	108.7	110.7
O2-V1-N11	146.2	144.9
O3-V1-O4	147.2	142.6
O3-V1-O5	104.7	105.4
O3-V1-N11	82.2	79.3
O4-V1-O5	102.7	103.7
O4-V1-N11	73.7	71.3
O5-V1-N11	104.2	103.7
V1-O4-C23	118.7	118.8
O4-C23-N8	117.6	117.3
O4-C23-N13	122.2	125.3
N8-C23-N13	120.2	117.3
C23-N13-N11	108.7	108.2
N13-N11-V1	115.6	115.5
N13-N11-C15	115.6	116.5
N11-C15-C14	122.9	123.3
C15-C14-C17	121.6	121.2
C15-C14-C24	119.9	121.0
C14-C17-O3	122.9	123.7
C17-O3-V1	135.9	133.7
C14-C17-C22	119.4	118.3
O3-C17-C22	117.7	117.9
C17-C22-C18	120.7	118.2
C17-C22-N12	121.5	122.9
C18-C22-N12	117.8	118.9
C22-N12-C25	119.0	118.3
N12-C25-C24	124.2	124.2
C25-C24-C27	118.5	118.9
C25-C24-C14	117.4	118.6
C24-C27-O6	113.3	113.3
R		0.993
MAE [°]		1.3

Table S3. Experimental and theoretical (at B3LYP-D3BJ/6-311++G(d,p)(H,C,N,O,S)/def2-TZVP(V) level of theory) bond lengths of **2** (the notation follows the figure below)

Bond length	Experimental	Theoretical
V1-O10	1.62	1.60
V1-O5	1.65	1.60
V1-O3	1.90	1.95
V1-N4	2.20	2.35
V1-S2	2.35	2.41
S2-C17	1.74	1.73
C17-N14	1.34	1.35
C17-N8	1.32	1.34
N8-N4	1.39	1.33
N4-C12	1.30	1.31
C12-C11	1.44	1.43
C11-C18	1.40	1.43
C18-O3	1.32	1.29
C18-C15	1.43	1.41
C15-C13	1.49	1.49
C15-N7	1.33	1.35
N7-C6	1.34	1.36
C6-C16	1.37	1.37
C16-C19	1.51	1.51
C19-O9	1.44	1.43
C16-C11	1.41	1.43
R		0.994
MAE [Å]		0.03

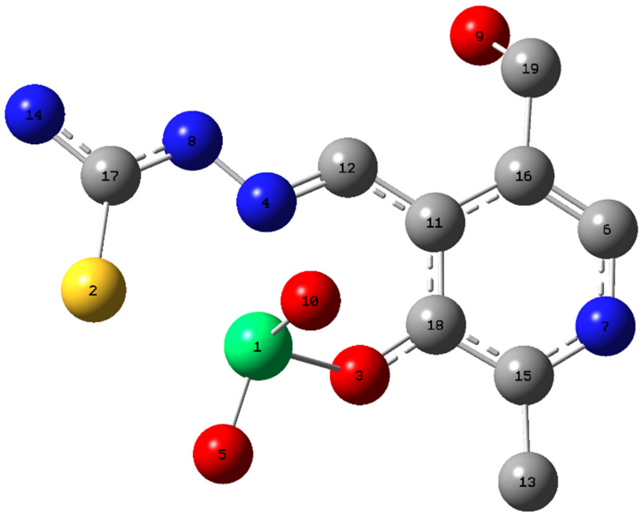

Table S4. Experimental and theoretical (at B3LYP-D3BJ/6-311++G(d,p)(H,C,N,O,S)/def2-TZVP(V) level of theory) bond angles of **2** (the notation follows the figure above)

Bond angle	Experimental	Theoretical
O10-V1-O5	108.9	106.6
O10-V1-O3	109.8	109.8
O10-V1-N4	98.2	93.9
O10-V1-S2	102.5	109.7
O5-V1-O3	97.4	99.4
O5-V1-N4	151.0	155.7
O5-V1-S2	86.9	91.3
O3-V1-S2	143.8	135.8
O3-V1-N4	82.5	77.8
S2-V1-N4	77.0	75.4
V1-S2-C17	100.1	100.6
S2-C17-N14	118.2	118.7
S2-C17-N8	123.7	126.5
C17-N8-N4	113.4	113.9
N8-N4-V1	121.8	121.5
N8-N4-C12	111.9	114.4
N4-C12-C11	125.4	123.3
C12-C11-C16	118.9	120.1
C12-C11-C18	121.7	121.6
C11-C18-O3	122.5	124.0
C18-O3-V1	134.8	128.2
C11-C18-C15	118.5	119.2
O3-C18-C18	119.0	116.8
C18-C15-C13	120.9	120.9
C18-C15-N7	120.1	119.1
C13-C15-N7	118.9	120.0
C15-N7-C6	121.0	123.5
N7-C6-C16	123.3	120.2
C6-C16-C11	117.7	119.8
C6-C16-C19	121.1	118.1
C16-C19-O9	109.2	113.4
C19-C16-C11	121.0	122.1
R		0.977
MAE [°]		2.5

Table S5. Experimental and theoretical (at B3LYP-D3BJ/6-311++G(d,p)(H,C,N,O,S)/def2-TZVP(V) level of theory) bond lengths of **3** (the notation follows the figure below)

Bond lengths	Experimental	Theoretical
V1-O4	1.64	1.60
V1-O5	1.63	1.61
V1-O3	1.92	1.95
V1-N9	2.20	2.30
V1-N7	2.00	2.04
O3-C15	1.31	1.29
C15-C16	1.41	1.41
C16-C17	1.49	1.49
C16-N10	1.34	1.35
N10-C18	1.35	1.36
C18-C19	1.37	1.37
C19-C20	1.50	1.51
C20-O6	1.43	1.43
C19-C14	1.42	1.43
C14-C15	1.41	1.43
C14-C13	1.44	1.42
C13-N9	1.30	1.31
N9-N8	1.38	1.33
N8-C11	1.34	1.35
C11-N7	1.32	1.31
C11-S2	1.75	1.77
S2-C12	1.79	1.82
R		0.996
MAE [Å]		0.02

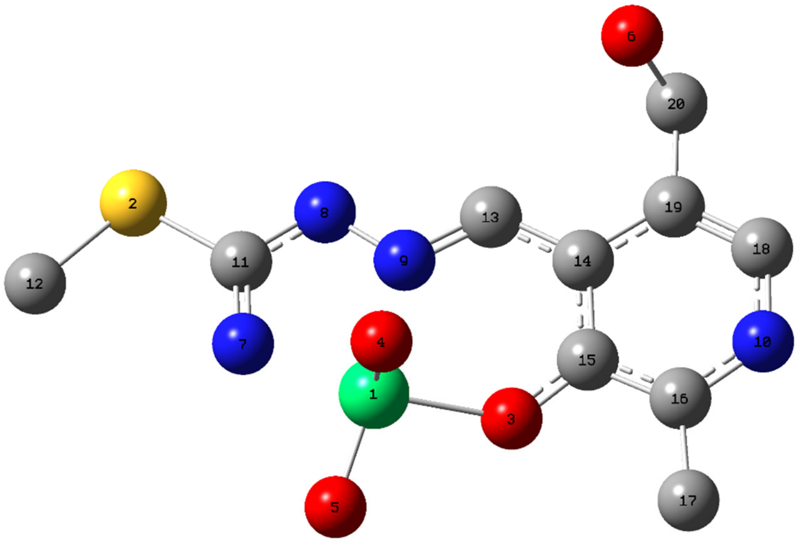

Table S6. Experimental and theoretical (at B3LYP-D3BJ/6-311++G(d,p)(H,C,N,O,S)/def2-TZVP(V) level of theory) bond angles of **3** (the notation follows the figure above)

Bond angle	Experimental	Theoretical
O4-V1-O5	108.1	110.4
O4-V1-O3	103.6	105.4
O4-V1-N9	105.0	101.1
O4-V1-N7	106.3	105.5
O5-V1-O3	96.8	99.7
O5-V1-N9	146.4	147.6
O5-V1-N7	92.8	93.7
O3-V1-N9	80.9	78.4
O3-V1-N7	143.9	139.4
N9-V1-N7	72.2	70.2
V1-O3-C15	134.1	131.7
O3-C15-C16	117.3	116.7
O3-C15-C14	123.9	124.2
C15-C16-C17	122.0	121.0
C15-C16-N10	118.8	119.1
C17-C16-N10	119.3	119.9
C16-N10-C18	123.8	123.5
N10-C18-C19	120.4	120.1
C18-C19-C20	119.4	118.2
C18-C19-C14	118.8	119.9
C19-C20-O6	109.6	108.6
C20-C19-C14	121.9	121.9
C19-C14-C15	119.4	118.2
C19-C14-C13	119.5	120.3
C15-C14-C13	121.0	121.4
C14-C13-N9	123.0	123.0
C13-N9-V1	129.1	126.3
V1-N9-N8	116.4	116.6
C13-N9-N8	114.4	116.1
N9-N8-C11	109.0	109.9
N8-C11-N7	121.5	122.3
C11-N7-V1	119.6	120.2
N8-C11-S2	110.9	111.8
C11-S2-C12	105.0	101.4
R		0.02
MAE [°]		1.4

Table S7. The important thermodynamic parameters (in kJ mol^{-1}) for the best docking conformation of the investigated complexes with BSA.

Compound	ΔG_{bind}	*Ki* (mM)	$\Delta G_{vdw+hbond+desolv}$	ΔG_{elec}	ΔG_{total}	ΔG_{tor}	ΔG_{unb}
**1**	-15.8	1.71	-17.1	-2.1	1.3	3.4	1.3
**2**	-19.3	0.41	-19.9	-2.8	0.9	3.4	0.9
**3**	-16.7	1.22	-16.0	-4.1	1.5	3.4	1.5
